# Complete Genome Sequences of *Microbacterium* Phages Clayda5 and Gshelby23 and *Gordonia* Phages Wrigley and Santhid

**DOI:** 10.1128/mra.00789-22

**Published:** 2022-10-10

**Authors:** Abigail M. Bastian, Marcus J. Blankespoor, Gideon K. Fynaardt, Sadie A. Gilmeister, Emily E. Hurley, Madison Jones, Erika J. McKenney, Alexa N. Olguin, Micah N. Rens, Garrett Snyder, Anneka E. Sterk, Sophie M. Swart, Alaena Trevino, Ashley N. Van Egdom, Morgan C. Veach, Kip Cullinan, Kaarina Van Berkum, Lauren R. Pavich, Krista Starr, Byron Noordewier, Sara S. Tolsma

**Affiliations:** a Department of Biology, Northwestern College, Orange City, Iowa, USA; Portland State University

## Abstract

Bacteriophages Clayda5, Gshelby23, Wrigley, and Santhid were isolated from soil samples collected in Iowa, with genomes typical of actinobacteriophages from clusters EB, EM, CY, and DY, respectively. Wrigley and Santhid were isolated on Gordonia terrae and are likely to be temperate. Clayda5 and Gshelby23 were isolated on Microbacterium foliorum.

## ANNOUNCEMENT

Bacteriophages that have been discovered and characterized are a tiny fraction of the plethora of these ubiquitous infectious particles on Earth (1). Here, we report the characteristics of four newly isolated actinobacteriophages, contributing to a growing understanding of bacteriophage diversity (2).

**FIG 1:**
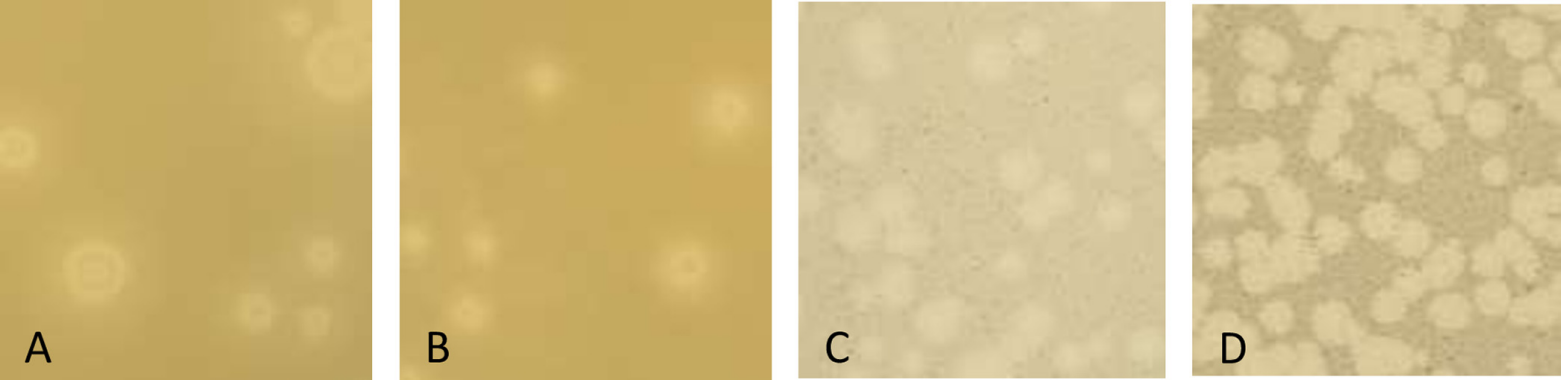
Plaque characteristics of phages Clayda5, Gshelby23, Santhid, and Wrigley after 48 h at 30°C. (A) Clayda5 yielded plaques that ranged from 1.5 to 5.5 mm in diameter (*n* = 10), with a mean diameter of 3.6 mm. (B) Gshelby23 yielded plaques that ranged from 1.6 to 4.8 mm in diameter (*n* = 10), with a mean diameter of 3.1 mm. (C) Santhid yielded plaques that ranged from 0.8 to 3.7 mm in diameter (*n* = 10), with a mean diameter of 2.4 mm. (D) Wrigley yielded plaques that ranged from 2.0 to 4.1 mm in diameter (*n* = 10), with a mean diameter of 3.2 mm.

The four actinobacteriophages were isolated from soil samples collected in Iowa using standard methods (3) (Table 1). Briefly, soil samples were washed with peptone-yeast extract-calcium (PYCa) liquid medium supplemented with 0.1% dextrose, and phages in the filtered wash (0.2-μm pore size) were isolated and purified with at least three rounds of plating in PYCa top agar overlays with either Microbacterium foliorum NRRL B-24224 (yielding phages Clayda5 and Gshelby23, which both form plaques with sharp, clear edges and a bullseye center) or Gordonia terrae 3512 (yielding phages Santhid and Wrigley, which both form slightly turbid plaques with less defined edges after 24 to 48 h at 30°C) (Fig. 1).

**TABLE 1:** Isolation details, sequencing details, and genomic characteristics of Clayda5, Gshelby23, Santhid, and Wrigley

Phage name	Soil sample collection site coordinates	Approximate shotgun coverage (×)	Genome size (bp)	Genome ends	G+C content (%)	No. of protein-coding genes	No. of tRNA genes	Cluster assignment
Clayda5	43.191844N, 96.138591W	601	39,894	3′ single-stranded overhang (5′-ACTCCCGGCA-3′)	67.2	72	2	EB
Gshelby23	42.6615N, 95.0975W	118	53,603	Circularly permuted	64.8	47	0	EM
Santhid	42.5924N, 96.0300W	1,126	39,295	3′ single-stranded overhang (5′-TCCGGAGGTA-3′)	67.7	60	0	DY
Wrigley	41.673144N, 93.725921W	1,445	51,878	3′ single-stranded overhang (5′-CGTATGGCAT-3′)	66.3	81	0	CY

Phage DNA was isolated from high-titer lysates using the Wizard DNA clean-up system (Promega) and then concentrated with the DNA Clean and Concentrator kit (ZYMO Research). DNA samples were pooled and then prepared for sequencing on an Illumina MiSeq sequencer using the NEBNext Ultra II FS kit, with each pool containing DNA from one *Gordonia* phage and one *Microbacterium* phage. Untrimmed reads were assembled using Newbler v2.9 and checked for completeness using Consed v29.0 with default parameters (4, 5). The genome ends were determined using PAUSE (https://cpt.tamu.edu/computer-resources/pause) and Consed v29.0 with default settings (4). The phage DNA sequences from each pooled sample were substantially different, which enabled the assembly of two distinct genomes (5). All phages were assigned to different clusters based on gene content similarity (GCS) of ≥35% with respect to sequenced bacteriophages present in the Actinobacteriophage Database, PhagesDB (6), using the PhagesDB GCS tool and previously described criteria (7). Sequencing details, genome characteristics, and cluster assignments are reported in Table 1.

The phage genomes were annotated using DNA Master v5.23.2 (http://cobamide2.bio.pitt.edu) with embedded Glimmer v3.02 and GeneMark v2.5, PECAAN (https://blog.kbrinsgd.org/about-us/), Phamerator, Starterator (http://phages.wustl.edu/starterator), HHpred, BLASTp, ARAGORN v1.1, and tRNAscan-SE v2.0, all with default parameters (8–14). We identified 72 protein-coding genes in Clayda5, 81 in Wrigley, 60 in Santhid, and 47 in Gshelby23 (Table 1). The genome of Clayda5 includes two tRNA genes (tRNA^Asp^ and tRNA^Glu^).

The genomes of both Wrigley and Santhid encode a putative tyrosine integrase (Wrigley_43 and Santhid_30, respectively) and a putative immunity repressor (Wrigley_49 and Santhid_31, respectively); therefore, these are likely temperate phages. A putative excise could be identified for Wrigley (Wrigley_55) but not for Santhid (15). We were able to identify a programmed translational frameshift (PTFS) in the tail assembly chaperone gene for Clayda5, Santhid, and Wrigley (16). Like other cluster EM phages, a slippery sequence characteristic of PTFSs could not be identified for Gshelby23. The genome of Wrigley encodes a protein (Wrigley_46) with predicted secondary and tertiary structure resembling that of SecB, the canonical bacterial export protein (17). The chemical characteristics of conserved residues for SecB are also conserved in Wrigley_46 (18). Finally, the right arm of the Wrigley genome diverges considerably from those of other phages in the CY cluster and contains three genes for which no homologs are present in PhagesDB (Wrigley_34, Wrigley_46, and Wrigley_48).

### Data availability.

The complete genome sequences of phages Clayda5, Gshelby23, Santhid, and Wrigley are available in GenBank (accession no. OM818329, ON260817, OM818327, and ON108651, respectively). The raw sequencing reads are available in the NCBI SRA under accession no. SRX14443493, SRX14443510, SRX14485115, and SRX14485106, respectively.
